# A 3-Week Inpatient Rehabilitation Programme Improves Body Composition in People with Cystic Fibrosis with and Without Elexacaftor/Tezacaftor/Ivacaftor Therapy

**DOI:** 10.3390/nu17152439

**Published:** 2025-07-25

**Authors:** Jana Koop, Wolfgang Gruber, Franziska A. Hägele, Kristina Norman, Catrin Herpich, Stefan Dewey, Christian Falkenberg, Olaf Schnabel, Burkhard Weisser, Mario Hasler, Anja Bosy-Westphal

**Affiliations:** 1 Institute of Human Nutrition and Food Science, Kiel University, Düsternbrooker Weg 17, 24105 Kiel, Germany; 2 Pediatric Pulmonology and Sleep Medicine, Cystic Fibrosis Center, Children’s Hospital, University of Duisburg-Essen, Hufelandstrasse 55, 45122 Essen, Germany; 3 Department of Nutrition and Gerontology, German Institute of Human Nutrition Potsdam-Rehbrücke, Arthur-Scheunert-Allee 114-116, 14558 Nuthetal, Germany; 4 Department of Geriatrics and Medical Gerontology, Charité-Universitätsmedizin Berlin, Reinickendorfer Strasse 61-62, 13347 Berlin, Germany; 5 Strandklinik St. Peter-Ording, Fritz-Wischer-Straße 3, 25826 St. Peter-Ording, Germany; 6 Fachklinik Satteldüne DRV Nord, Tanenwai 32, 25946 Nebel, Germany; 7 Department of Sportsmedicine, Kiel University, Olshausenstrasse 74, 24118 Kiel, Germany; 8 Applied Statistics, Kiel University, Hermann-Rodewald-Strasse 9, 24098 Kiel, Germany

**Keywords:** triple CFTR modulators, Elexacaftor/Tezacaftor/Ivacaftor, appetite control, energy balance, CF rehabilitation, body composition

## Abstract

Background: The introduction of cystic fibrosis transmembrane conductance regulator modulators, especially the triple therapy elexacaftor, tezacaftor, ivacaftor (ETI), has improved outcomes in people with cystic fibrosis (pwCF), reducing underweight but increasing overweight rates. Objectives: This study investigates the effect of ETI on appetite control, body composition, and energy balance during a 3-week inpatient rehabilitation programme with regular exercise. Methods: In 54 pwCF (38 on ETI, 16 without ETI), changes in body composition (fat mass index, FMI; fat-free mass index, FFMI) and energy balance (calculated from body composition changes) were assessed. Appetite control was evaluated via plasma peptide YY (PYY) levels and post-exercise meal energy intake. Results: The programme significantly increased BMI (+0.3 ± 0.1 kg/m^2^; CI 0.1–0.4) and energy balance (+4317 ± 1976 kcal/3 weeks), primarily through FFMI gains (+0.3 ± 0.1 kg/m^2^; CI 0.1–0.4). Despite higher post-exercise meal energy intake and a tendency towards lower PYY levels in the ETI group, changes in body composition and energy balance did not differ between groups. This is explained by a higher prevalence of exocrine pancreatic insufficiency in the ETI group (92% vs. 50%, *p* < 0.001). Small sample sizes limit the interpretation of data on appetite control and energy intake. Conclusions: A 3-week inpatient rehabilitation programme improved body composition in pwCF, without resulting in a more positive energy balance with ETI therapy. This is due to a higher prevalence of pancreatic insufficiency in this group.

## 1. Introduction

Cystic fibrosis (CF) is an autosomal recessive multiorgan disease caused by mutations of the cystic fibrosis transmembrane conductance regulator (CFTR) gene, which leads to impaired CFTR channel function and compromised chloride and bicarbonate secretion [1]. This results in thickened mucus secretions that affect the respiratory and gastrointestinal tracts [1]. Organ manifestations include reduced lung function and exocrine pancreatic insufficiency [1], which affect about 90% of people with CF (pwCF) in Germany [2]. Patients have a reduced life expectancy compared to the general population [1]. CF used to be associated with malnutrition and underweight that were linked to a worse prognosis [3,4].

The introduction of CFTR modulators in Europe in 2012 [5] has led to marked improvements in patient outcomes [6,7]. These agents target the CFTR protein defect by potentiating (ivacaftor) or correcting (elexacaftor, tezacaftor, lumacaftor) its function [7] and are used in 83% of adults with CF in Germany [2]. The introduction of CFTR modulators significantly contributed to reducing the proportion of pwCF who were underweight from 18% to 7% between 2000 and 2023 [2]. However, concomitantly, the prevalence of overweight and obesity increased from 8% to 23% [2], particularly among individuals receiving the triple combination of elexacaftor, tezacaftor, and ivacaftor (ETI) [8,9], approved in 2020 in Europe [10]. A decrease in resting energy expenditure [8], potentially due to improved lung function and reduced inflammation [11], and/or a higher energy intake [8] may contribute to a higher risk of body weight gain in pwCF receiving ETI therapy [8].

A great number of studies on the impact of ETI on body composition have demonstrated a disproportionate gain in fat mass (FM) following the initiation of ETI treatment in adults with CF [12,13,14,15,16,17,18,19,20]. Conversely, a smaller number of studies have reported an appropriate increase in both FM and fat-free mass (FFM) [21,22,23]. While an improvement in FFM is strongly associated with better pulmonary function [24,25] and thus better outcomes in pwCF, the combination of high FM with low FFM, which can be masked by a high body mass index (BMI), has been linked to worse lung function compared with those with better FFM [25]. The rising prevalence of overweight and obesity may also lead to a higher risk of non-communicable diseases such as cardiovascular disease [25].

It is conceivable that ETI treatment may exert an influence on two distinct yet interrelated aspects of energy metabolism: (i) the absolute energy balance, which may involve a reduction in resting energy expenditure or stimulation of appetite, and (ii) energy partitioning, which affects the distribution of gains in FM and FFM.

In Germany, inpatient rehabilitation programmes for pwCF are traditionally directed towards weight gain and aim to improve body composition, exercise capacity, and overall health with a structured, multidisciplinary approach that includes nutrition therapy, physiotherapy, and regular exercise [26,27]. It is recommended that pwCF engage in regular physical activity (30–60 min daily of moderate-to-vigorous and resistance training 2–3 times per week), as research has demonstrated that regular exercise can enhance lung function and quality of life, while concurrently reducing pulmonary exacerbations and hospitalisation [28]. The observed increase in weight during inpatient rehabilitation programmes [27] could partly be due to an appetite-stimulating effect of the regular training sessions in the programme [29]. Given that ETI therapy already predisposes pwCF to weight gain [25], it is conceivable that ETI treatment during an inpatient rehabilitation programme could result in a more positive energy balance compared to pwCF without CFTR modulator treatment.

We therefore investigated the effect of ETI treatment on appetite control, changes in body composition, and energy balance in pwCF during a 3-week inpatient rehabilitation programme comprising an exercise intervention. PwCF without CFTR modulator therapy served as a control (non-ETI). It was postulated that individuals who received ETI treatment exhibit a greater post-exercise appetite (higher subjective appetite and lower plasma levels of peptide YY, PYY) and energy intake (meal post-exercise and 24 h energy intake) as well as a more positive energy balance (calculated from changes in body composition and accompanied by an increase in plasma leptin levels). This secondary analysis provides novel insights into the effects of triple CFTR modulator therapy on changes in body composition and appetite control during inpatient rehabilitation programmes. To the best of our knowledge, this is an area that has not been studied before, yet it is highly relevant to clinical practice given the increasing prevalence of overweight and obesity in this population.

## 2. Materials and Methods

### 2.1. Study Protocol and Study Population

This is a secondary analysis that is based on a study that investigated the effect of two training programmes with different modalities (high-intensity interval training, HIIT, and moderate-intensity continuous training, MICT) on exercise capacity, body composition, and appetite control in pwCF as a primary aim [30]). The trial was registered at clinicaltrials.gov as NCT05140967. The study was conducted in accordance with the guidelines outlined in the Declaration of Helsinki, and all procedures involving human subjects were approved by the Ethics Committee of the Medical Faculty at Kiel University, Kiel, Germany (D 588/21). Written informed consent was obtained from all subjects. The study was conducted with an identical protocol in two inpatient rehabilitation clinics (Fachklinik Satteldüne, Nebel, Amrum, Germany and Strandklinik St. Peter-Ording, St. Peter-Ording, Germany) as part of a 3-week rehabilitation programme for pwCF, including a training intervention.

Between January 2022 and December 2023, 70 pwCF were recruited to participate in the study. Inclusion criteria comprised a confirmed diagnosis of CF by at least two sweat tests and/or by the presence of two CF mutations, age ≥ 18 years, and percent predicted forced expiratory volume in one second (ppFEV1) ≥ 40%. Exclusion criteria were acute pulmonary exacerbation in the 4 weeks before the programme, cor pulmonale, pulmonary hypertension, or musculoskeletal complaints that make regular physical activity impossible. Prevalence of exocrine pancreatic insufficiency, CF-related diabetes (CFRD), genotype, Pseudomonas aeruginosa infection, and CFTR modulator intake were obtained from the medical records of the participants.

**Figure 1:**
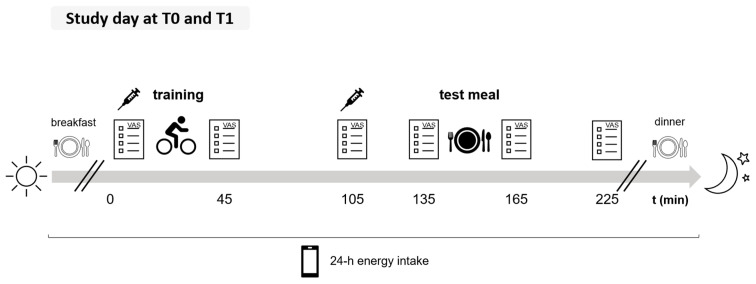
Study protocol at baseline (T0) and at the end (T1) of the 3-week rehabilitation programme. Participants had breakfast, followed by a training session (High-Intensity Interval Training or Moderate-Intensity Continuous Training) on a cycle ergometer for 30 min. Meal energy intake post-exercise was assessed with an ad libitum test meal (pasta dish) 90 min after the end of the training session. Subjective appetite ratings were obtained using visual analogue scales (VASs) at six time points: before and after exercise, 60 min post-exercise, before and after the test meal, and 60 min post-meal. Appetite-regulating hormones were measured in blood samples taken before training (plasma peptide YY, PYY, and plasma leptin) and 60 min after training (plasma PYY). Participants documented 24 h energy intake with smartphone photos and written entries using a web application (Meal-Tracking Web App, Institute of Human Nutrition, Kiel University, Kiel, Germany).

Outcome parameters, which were also evaluated in this analysis, were measured at baseline (T0) and the end (T1) of the intervention and included BMI, body composition (fat mass index, FMI; fat-free mass index, FFMI; both kg/m^2^), and the energy balance calculated from changes in FM and FFM. Post-exercise subjective appetite, appetite-regulating hormone concentrations (plasma levels of PYY and leptin), and meal energy intake 90 min after a training session, as well as 24 h energy intake, were also assessed at T0 and T1. The two study days, at baseline (T0) and the end of the rehabilitation programme (T1), were conducted following the protocol outlined in Figure 1.

In the original study, participants were randomised to either HIIT or MICT. Since the type of training did not affect the outcome parameters, the participants could then be categorised into two groups for this secondary analysis: those with ETI therapy and those who did not receive ETI or other CFTR modulators (non-ETI group).

### 2.2. Training Protocol

In the initial study, participants were randomised based on their lung function (ppFEV < 70% or ppFEV ≥ 70%) to either HIIT (10 × 1 min at 90% peak oxygen uptake, VO_2peak_ with 2 min active rest after each interval at 40% VO_2peak_, based on cardiopulmonary exercise testing) or MICT (60% of VO_2peak_), both performed on a cycle ergometer 3 times per week for 30 min in the morning after breakfast. All training sessions were supervised by a sports therapist or physiotherapist. Participants also completed one weekly strength training session and one weekly session of various sports activities (e.g., gymnastics or yoga), totalling five exercise sessions per week. Since the primary aim of the study was to improve exercise capacity, the training was predominantly aerobic.

### 2.3. Spirometry

Lung function was assessed using body plethysmography (Jaeger^®^ MasterScreen Body, Vyaire Medical, Hoechberg, Germany), and reference values for ppFEV1 were calculated according to the Global Lung Function Initiative Reference System [31]. VO_2peak_ was determined using cardiopulmonary exercise testing performed at baseline (T0) on a cycle ergometer (ergoselect 200, ergoline, Bitz, Germany), following the Godfrey protocol [32,33]. Gas exchange measurements were recorded breath-by-breath (Vyntus TM CPX, Vyaire Medical, Hoechberg, Germany), and VO_2peak_ was defined as the highest value in the last 30 s of the test.

### 2.4. Subjective Appetite

Subjective appetite ratings were obtained using horizontal 100 mm visual analogue scales (VAS; 0 mm: ’not at all’, 100 mm: ‘extremely’) [34]. Participants reported their perceptions of hunger, fullness, desire to eat, and prospective food consumption at several time points (Figure 1): before and immediately after training, 60 min after the end of the training, immediately before and after the test meal, and 60 min post-meal. The appetite score was calculated as the mean of all appetite ratings (inverse of fullness) for all time-points [35,36]. The Satiety Quotient (SQ) was calculated for two time points immediately after the test meal and 60 min after the end of the test meal, using the following formula according to Drapeau et al. (2007) [37] and Green et al. (1997) [38].

SQ (mm/kcal) = [pre-meal rating of hunger (mm) − post-meal rating of hunger (mm)]/energy content of the test meal (kcal)] × 100

A higher SQ value indicates a greater reduction of hunger per calorie, demonstrating a greater satiating effect of the meal [38]. Additionally, participants were required to rate the palatability and pleasantness of the test meal using a VAS.

### 2.5. Appetite-Regulating Hormones

Participation in the collection of blood samples was voluntary. If consent was given, blood samples (EDTA plasma) were drawn before and 60 min after the end of the training session at T0 and T1 (Figure 1). As breakfast is part of the standard routine in the rehabilitation clinic, and to ensure safety and effectiveness of the morning exercise session, participants were not fasting before blood sample collection. Samples were centrifuged directly at 2500× *g* for 10 min and stored at −80 °C until analyses. Concentration of total plasma PYY (ng/mL) was analysed using a human ELISA-based assay (intra-assay CV: 6.1–8.5%, inter-assay CV: 5.5–10.3%, Yanaihara Institute Inc., Shizuoka, Japan). Plasma leptin concentration (ng/mL) was assessed in pre-training samples at T0 and T1 using a human ELISA-based assay (intra-assay CV of 4.2% and an inter-assay CV of 6.7%; BioVendor, Brno, Czech Republic). All analyses were conducted according to the manufacturer’s instructions at the ‘German Institute of Human Nutrition’ (Department of Nutrition and Gerontology, Potsdam-Rehbrücke, Nuthetal, Germany).

### 2.6. Energy Intake

The meal energy intake post-exercise was assessed with a test meal 90 min after the end of the training session on both study days (Figure 1). Subjects received a standardised lunch consisting of a pasta dish with 65% carbohydrates (CHO), 13% protein, and 22% fat. The energy content of the initial portion was 25% of the participants’ resting energy expenditure (REE; Harris & Benedict, 1918 [39]) and was weighed accurately to the gram using an electronic kitchen scale (Soehnle, Nassau, Germany). Depending on their appetite, subjects could receive additional portions (large: 25% kcal/REE; small: 12.5% kcal/REE). They ate ad libitum and were required to finish the meal within 30 min. To avoid distracted eating, the use of media or conversations with other participants were not allowed. Subjects received a glass of water (150 mL) that had to be consumed during the meal. The amount eaten of the test meal was recorded by weighing the leftovers accurately to the gram.

At T0 and T1, participants documented their 24 h energy intake (including breakfast, dinner, snacks, and caloric beverages) with smartphone photos and written entries, using a web application specifically developed for study purposes by the Institute of Human Nutrition (Meal-Tracking Web App, Kiel University, Kiel, Germany). The data on energy intake at the test meal were added by the study staff for the analysis of 24 h energy intake. Energy intake (% kcal/REE) and amounts of CHO, fat (% of calories), protein (% of calories, and g/kg body weight), sugar (% of calories), and salt (g/day) were analysed using PRODI expert version 6.12 (Wissenschaftliche Verlagsgesellschaft, Stuttgart, Germany). Percentages of macronutrients (% of calories) were calculated using the following factors: protein (4 kcal/g), CHO (4 kcal/g), and fat (9 kcal/g) [40].

### 2.7. Body Composition and Energy Balance

Height was measured at baseline, using a stadiometer (seca 274, seca Hamburg, Germany). Body weight was assessed on an electronic scale at T0 and T1 (seca 861, seca Hamburg, Germany). Body composition (FM, FFM) was determined using bioelectrical impedance analysis (seca 515; seca 525, Hamburg, Germany) at T0 and T1. BMI, FMI, and FFMI were calculated as kg/m^2^. Given known physiological differences in body composition between males and females, FMI and FFMI were analysed separately by sex, as the initial body fat content is a determining factor for the relative contribution of FFM and FM to total body weight change [41].

Energy balance was calculated based on the measured changes in body composition (T1–T0), using the following formula, which incorporates coefficients for tissue gains (covering energy content of the tissue plus energy costs for tissue synthesis) [42], as well as coefficients for tissue losses [43,44].

Energy balance (kcal/3 weeks) = (13.1 × FM gain, g) + (2.2 × FFM gain, g) − (9.3 × FM loss, g) − (1.1 × FFM loss, g)

Positive values indicated a positive energy balance and vice versa.

### 2.8. Statistical Analysis

A priori power analysis for the original study (‘Effect of HIIT vs. MICT on exercise capacity, body composition, and appetite control in pwCF’) was performed using G*Power version 3.1.9.7 [45], based on the results of a study conducted by Gruber et al. (2014) [46]. To detect a 10% difference (SD: 15%) in the primary outcome (VO_2peak_) with 80% power and an alpha level of 0.05, a total of 28 participants per group (HIIT or MICT) were required. To account for a 20% dropout, 70 participants (35 per group) were enrolled.

The statistical software R version 4.4.1 (2024) was used to evaluate the data [47]. The analysis started with comparisons of baseline characteristics between ETI and non-ETI groups using independent *t*-tests (age, height, REE, ppFEV1, BMI, FMI, FFMI) and chi-squared tests for categorical variables (training type, sex, pancreatic insufficiency, CFRD, Pseudomonas aeruginosa, genotypes). It was followed by the definition of appropriate statistical models, a linear mixed model [48] and a model based on generalised least squares [49]. The linear mixed model included the fixed factors medication (ETI, non-ETI), training type (HIIT, MICT), sex (female, male), and time point (T0, T1), along with all their interaction terms as fixed factors and subject ID as a random factor. The model based on generalised least squares did not include the fixed factor time point and the random factor subject ID. It was used to compare increases/decreases for the variables ΔBMI, ΔFMI, ΔFFMI, Δplasma leptin, and energy balance between the ETI and non-ETI groups.

Potential confounders, including exocrine pancreatic insufficiency and FMI at T0 (for energy balance, ΔBMI, ΔFMI, ΔFFMI, and Δplasma leptin) and palatability (for meal energy intake post-exercise), were tested and included as covariates if *p* < 0.1. Due to the association of exocrine pancreatic insufficiency with poor nutritional status [50], comparisons between the ETI and non-ETI groups regarding energy balance, ΔBMI, ΔFMI, ΔFFMI, and Δplasma leptin were adjusted for the prevalence of pancreatic insufficiency.

The residuals were assumed to be normally distributed and heteroscedastic (if necessary), with graphical residual analysis to confirm. Based on the model, an ANOVA was conducted, followed by appropriate multiple contrast tests [51,52]. The significance of increases/decreases in the variables ΔBMI, ΔFMI, ΔFFMI, energy balance, Δplasma leptin, and PYY levels was assessed with simultaneous 95% CIs. Results comparing ETI vs. non-ETI were averaged across training type and time points, unless otherwise stated. The unequal sample sizes between the two groups were accounted for by the linear (mixed) model and multiple contrast tests. Participants with a missing value were excluded from the statistical analysis, separately for each measurement variable. Data presented as boxplots show the median (horizontal line within the box) and the IQR (box limits represent the 25th and 75th percentiles). Whiskers extend to 1.5 times the IQR, and the mean value is indicated by the ‘x’. Results are presented as mean ± SE and 95% CI. Significance was set at *p* < 0.05. Figures were created using Microsoft^®^ Excel (version 16.95.1), Microsoft^®^ PowerPoint (version 16.95.1), and GraphPad Prism 10 (version 10.4.1).

## 3. Results

A total of 70 participants were recruited for the study. The dropout rate was 14.3% (10 participants, ETI: *n* = 4; non-ETI: *n* = 6), resulting from premature cessation of participation or illness among the participants. Two subjects were excluded from the subsequent analysis, as they were receiving mono- or dual CFTR modulator therapy. Missing data for outcome parameters (due to unsuccessful blood draws, incomplete questionnaires, and organisational problems) are shown in Table S1.

### 3.1. Baseline Characteristics

Comparison of baseline characteristics between ETI and non-ETI groups is shown in Table 1. The proportion of participants in the different types of training did not differ between the groups (HIIT/MICT, ETI: 47/53%, non-ETI: 44/56%; *p* = 0.89). The ETI vs. non-ETI group exhibited a higher prevalence of exocrine pancreatic insufficiency, as well as a greater incidence of homozygous and heterozygous Delta F508 genotypes. No further differences in baseline characteristics were found between the groups. According to WHO criteria, no participants were classified as being underweight, 38 participants had normal weight (ETI: *n* = 29; non-ETI: *n* = 9), 13 participants were overweight (ETI: *n* = 8; non-ETI: *n* = 5), and 3 participants had obesity (ETI: *n* = 1; non-ETI: *n* = 2) [53]. The age range was 19–57 years for the ETI group and 22–56 years for the non-ETI group. Training type did not affect the results between ETI and non-ETI groups in all outcome parameters.

### 3.2. Exercise-Induced Appetite and Energy Intake

**Figure 2:**
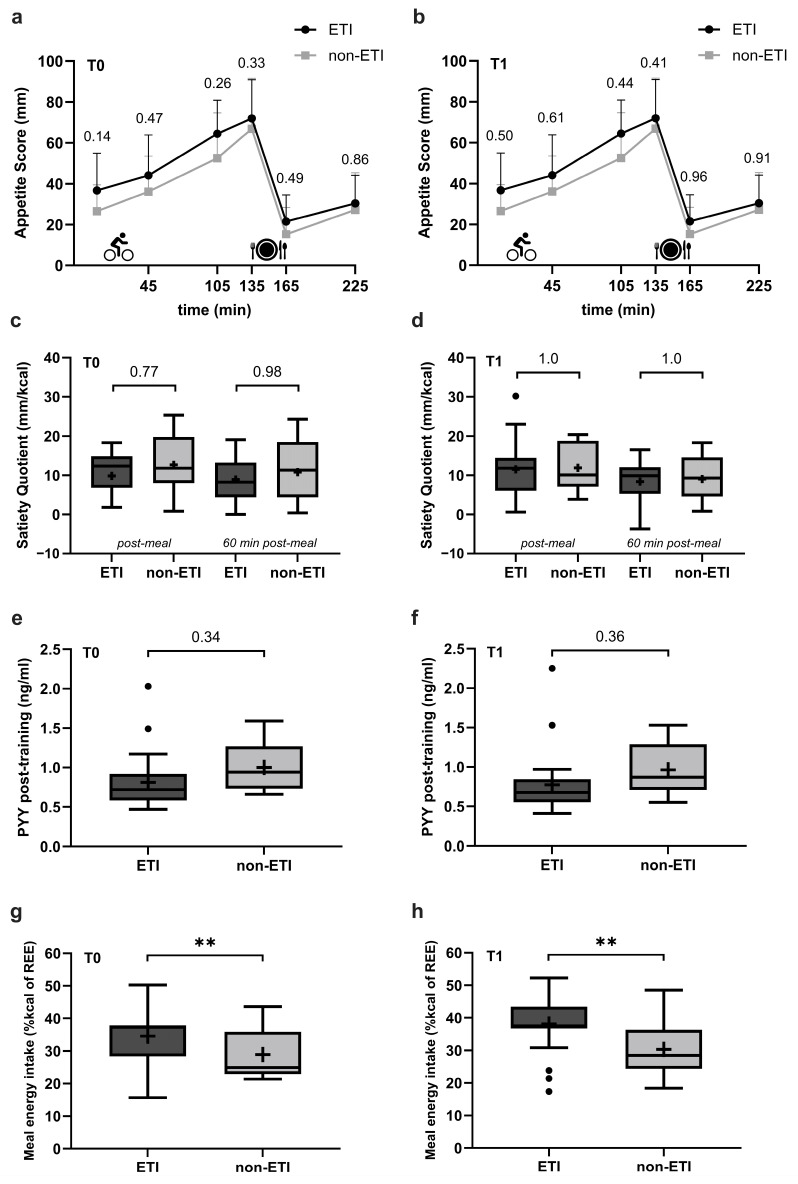
Comparison of appetite scores, satiety quotients, plasma PYY concentrations, and post-exercise meal energy intake between the ETI group (Elexacaftor/Tezacaftor/Ivacaftor therapy) and the non-ETI group (no CFTR modulator therapy) at baseline (T0) and at the end (T1) of the rehabilitation programme. Appetite scores (**a**,**b**) (mean of subjective appetite ratings of hunger, fullness, desire to eat, and prospective food consumption) were assessed using visual analogue scales at six time points: before and after exercise (30 min on cycle ergometer), 60 min post-exercise, before and after the ad libitum test meal, and 60 min post-meal. Satiety quotients (**c**,**d**) show the change in hunger ratings before vs. immediately after or 60 min after the test meal in relation to the energy content of the meal (kcal). Plasma PYY concentrations (**e**,**f**) were analysed from blood samples taken 60 min post-exercise. Post-exercise meal energy intake (**g**,**h**) 90 min after the exercise, expressed as a percentage of calculated resting energy expenditure according to Harris and Benedict (1918) [39]. (**a**–**d**): ETI, *n* = 22; non-ETI, *n* = 11. (**e**,**f**): ETI, *n* = 24; non-ETI, *n* = 12. (**g**,**h**): ETI, *n* = 25; non-ETI, *n* = 12. REE, resting energy expenditure; PYY, peptide YY. Results are adjusted for training type and sex. Dots indicate data points identified as outliers. *p*-values indicate differences between the ETI and non-ETI groups assessed by contrast tests (based on linear mixed model), ** *p*  <  0.01.

In the total sample, no differences in appetite scores (pre- and post-exercise) and SQ were observed between the ETI and non-ETI groups at baseline and the end of the inpatient rehabilitation programme (Figure 2a–d).

Plasma PYY levels did not differ between ETI and non-ETI groups at T0 and T1 before training (T0, ETI: 0.9 ± 0.1, non-ETI: 1.1 ± 0.1 ng/mL; *p* = 0.25; T1, ETI: 0.8 ± 0.1, non-ETI: 1.0 ± 0.1 ng/mL, *p* = 0.29) and 60 min after training (T0, ETI: 0.8 ± 0.1, non-ETI: 1.0 ± 0.1 ng/mL; *p* = 0.34; T1, ETI: 0.8 ± 0.1, non-ETI: 0.9 ± 0.1 ng/mL, *p* = 0.36) (Figure 2e,f). When averaged over T0 and T1, plasma PYY concentration was lower in the ETI compared to the non-ETI group pre-training (ETI: 0.9 ± 0.05, non-ETI: 1.1 ± 0.1 ng/mL, *p* = 0.04) and tended to be lower 60 min after the training session (ETI: 0.8 ± 0.05, non-ETI: 1.0 ± 0.1 ng/mL, *p* = 0.07). In all participants, each training session resulted in a significant reduction in PYY levels (T0, 95% CI −0.1–(−0.1); T1: 95% CI −0.1–(−0.05)).

Meal energy intake post-exercise was higher in the ETI group than in the non-ETI group both at baseline and at the end of the inpatient rehabilitation programme (Figure 2g,h). The pleasantness of the test meal was rated similarly between ETI and non-ETI groups (66.2 ± 4.3 vs. 62.3 ± 7.1 mm; *p* = 0.64), but the palatability tended to be rated higher in the ETI vs. the non-ETI group (66.0 ± 3.8 vs. 52.4 ± 6.2 mm; *p* = 0.07).

Self-reported 24 h energy intake tended to be higher in the ETI vs. non-ETI group (163.7 ± 9.1% vs. 133.0 ± 13.0% kcal/REE; *p* = 0.06). The 24 h protein intake was significantly higher in the ETI compared to the non-ETI group (1.3 ± 0.1 vs. 1.0 ± 0.1 g/kg body weight; *p* = 0.008).

### 3.3. Changes in Body Composition, Energy Balance, and Plasma Leptin Concentrations

A significant increase in BMI and energy balance with the rehabilitation programme was observed in the total group of pwCF (BMI: +0.3 ± 0.1; 95% CI 0.1–0.4 kg/m^2^; energy balance: +4317 ± 1976, 95% CI 317–8316 kcal/3 weeks). Subgroup analysis showed that the increase in BMI was evident in all groups except for the non-ETI group participating in HIIT (+ 0.1 ± 0.2; 95% CI −0.3–0.6 kg/m^2^). The positive energy balance was also true for the subgroup of women (6936 ± 2374, 95% CI 2130–11,741 kcal/3 weeks) but not for men (1697 ± 3052, 95% CI −4480–7875 kcal/3 weeks). The increase in energy balance in the total sample was due to an increase in FFMI (+0.3 ± 0.1, 95% CI 0.1–0.4 kg/m^2^), whereas FMI remained unchanged (−0.01 ± 0.1, 95% CI −0.2–0.2 kg/m^2^). In the total sample, pwCF with exocrine pancreatic insufficiency had a lower energy balance compared to pancreatically sufficient pwCF (−629 ± 2327 vs. +9262 ± 3314 kcal/3 weeks; *p* = 0.01). Analysis of subgroups of pwCF with or without exocrine pancreatic insufficiency within ETI or non-ETI groups showed that only those with sufficient pancreatic function demonstrated a positive energy balance following the intervention (Table S2).

Table 2 shows the comparison of 3-week changes in BMI, body composition, energy balance, and plasma leptin levels between the ETI and non-ETI groups, adjusted for the prevalence of exocrine pancreatic insufficiency and training type. No between-group differences were observed in all these parameters. In the ETI group, women demonstrated a more positive energy balance than men (*p* = 0.03).

## 4. Discussion

The inpatient rehabilitation programme resulted in an increase in BMI and energy balance in the total group of pwCF, with no differences between ETI and non-ETI treatment. However, plasma PYY levels tended to be lower in the ETI group compared to the non-ETI group (averaged over T0 and T1), and post-exercise meal energy intake (in both men and women) was higher in the ETI group. This discrepancy is explained by a higher prevalence of exocrine pancreatic insufficiency in the ETI group. This is supported by the observation of a lower energy balance in pwCF and pancreatic insufficiency when compared with those pwCF with sufficient pancreatic function (see results). In addition, analysis of subgroups of pwCF with or without pancreatic insufficiency within ETI or non-ETI groups revealed that only pancreatically sufficient pwCF had a positive energy balance due to the intervention (Table S2). It is expected that pancreatic insufficiency impairs energy balance, given that it results in malabsorption of macronutrients [50]. Notably, the non-ETI group exhibited a lower prevalence of pancreatic insufficiency compared to the average CF population in Germany (89.5%) [2]. At the time of the study, ETI was approved for the treatment of pwCF with at least one ΔF508 mutation in the CFTR gene [10]. This mutation, along with other severe genotypes such as G551D and R553X, is associated with greater disease severity, including exocrine pancreatic insufficiency, impaired pulmonary function, and increased mortality [54]. Severe alleles (ΔF508, G551D, and/or R553X) [54] were found in 47% in the ETI and 25% in the non-ETI group.

A systematic review demonstrated that CFTR modulators such as ivacaftor improved exocrine pancreatic function in pediatric patients [55], but data on the effects of ETI on exocrine pancreatic insufficiency in adults remain limited. One study reported reduced pancreatic enzyme replacement therapy requirements in adults on ETI therapy [56], and another found that weight gain after one year of ETI treatment occurred only in those with exocrine pancreatic insufficiency [57]. However, given the limited evidence, the extent to which ETI improves exocrine pancreatic insufficiency and thereby contributes to weight gain in adults with CF remains uncertain.

A further aspect is that ETI treatment has been observed to stimulate appetite [58] and thus energy intake [59], whereas other authors found that the increase in body weight with ETI was not due to increased energy intake [60]. The lower plasma PYY concentrations in the ETI group compared to the non-ETI group (only when averaged over T0 and T1) suggest a higher appetite in participants with ETI therapy [61]. Taste and smell are frequently impaired in pwCF due to sinus disease, which could explain the poor energy intake during exacerbations of illness [62]. While some studies have reported that ETI therapy can improve olfaction [63,64], other studies have shown no significant changes after the initiation of ETI treatment [65,66]. Potential mechanisms regarding the effects of ETI on olfaction are still unclear, but improvements in mucociliary clearance through enhanced mucus hydration and reduced sinonasal inflammation [63] may contribute to better olfactory function. The ETI group in this study rated the palatability of the test meal more highly than the non-ETI group (see Results). Although the higher ratings of palatability may account for the increased meal energy intake observed in the ETI group, group differences remained evident after adjusting for palatability (ETI: 36.8 ± 1.7 vs. non-ETI: 28.6 ± 2.7% kcal/REE; *p* = 0.02).

The intake of fat and CHO between ETI and non-ETI groups did not differ, but a higher protein intake was found in pwCF with ETI therapy (+0.3 g/kg body weight). A high protein intake is known to increase satiety [67] and thereby usually decreases energy intake, both acutely [68,69] and in the long-term [70], but a secondary analysis of studies in healthy individuals found a positive association between energy intake and protein content in contemporary diets with moderate levels of CHO (48%) and fat (37%) [71]. These authors suggested that other dietary factors, such as a high energy density and hyper-palatability of the foods, contributed to the observed higher energy intake with a higher protein intake [71]. The regular exercise sessions in the rehabilitation programme (5 × 45 min/week) likely contributed to a limitation of a positive energy balance during the rehabilitation programme (on average +206 kcal/d), as higher energy turnover facilitates weight maintenance due to appetite sensations that better correspond to energy requirements [72].

Changes in body composition were comparable between the ETI and non-ETI groups, and no disproportionate increase in BMI or FMI was observed in the ETI group (Table 2). This may be due to the beneficial effect of the exercise programme on energy partitioning, which led to an increase in FFMI while FMI remained unchanged (Table 2). The relatively high protein intake of the participants (>1 g/kg body weight in both groups, see Results) could also have contributed to the observed increase in FFMI. The type of training did not affect the results of comparisons of energy balance, changes in body composition, and BMI in the ETI vs. non-ETI groups, however, a subgroup analysis showed that an increase in BMI was evident in all groups except for the non-ETI group participating in HIIT (0.1 ± 0.2; 95% CI −0.3–0.6 kg/m^2^).

An increase in FFM while maintaining FM was also observed in individuals with severe CF during an inpatient rehabilitation programme with a comparable exercise intervention [46], as well as in an outpatient setting with three weekly exercise sessions [73], both over a period of 6 to 8 weeks. In contrast, a gain in both FMI and FFMI was found in pwCF with low BMI during a 3-week rehabilitation programme with daily exercise sessions [27]. The relevance of FFM for pwCF is evidenced by its association with better respiratory outcomes [25]. Additionally, regular exercise in pwCF is linked to better physical fitness and thus a better prognosis [74,75]. Therefore, the recently published ‘ESPEN-ESPGHAN-ECFS guideline on nutrition care for cystic fibrosis’ emphasises the importance of regularly monitoring body composition and recommends regular physical activity to increase FFM or prevent its loss [25]. Our findings show that rehabilitation programmes should prioritise improving body composition over body weight. In particular, those with less severe pancreatic insufficiency would benefit from exercise programmes that improve energy partitioning towards an increase in FFM rather than FM. This aspect appears to be highly significant given that, in April 2025, the European Medicines Agency decided to extend the indication for ETI therapy to all pwCF who have at least one non-Class I mutation in the CFTR gene [10]. Consequently, people with less severe pancreatic insufficiency will also receive ETI therapy in the future.

A limitation of this study was the lack of data regarding the duration of prior CFTR modulator use or ETI therapy. Therefore, we could not evaluate the effect of the duration of ETI therapy on weight gain. Although weight gain has been shown to be most pronounced at the first phase of ETI therapy [76], inpatient rehabilitation programmes in pwCF usually achieve weight gain as a therapeutic target [26,27]. According to our hypothesis, if the ETI group is indeed at a higher risk of weight gain, they should have gained more weight during the rehabilitation programme, even if they had been taking the medication for a long time. If the duration of previous ETI therapy had been shorter, weight gain in this group would have been even higher. Other limitations are the small number of participants, the short duration of the intervention, potential differences in the severity of the disease (e.g., exocrine pancreatic insufficiency) between groups, and unequal group sizes. Even though we accounted for unequal sample sizes by the linear (mixed) model and multiple contrast tests, future studies need to confirm the finding of equal weight gain in ETI and non-ETI groups during rehabilitation programmes. Further, studies with larger sample sizes and a higher age range need to investigate age and sex differences in the regulation of energy balance.

## 5. Conclusions

The 3-week inpatient rehabilitation programme for pwCF, including five exercise sessions per week, led to an increase in BMI attributed solely to a gain in FFMI, independent of ETI therapy. Although participants in the ETI group reported a greater palatability of the food, exhibited lower plasma PYY levels, and had a higher energy intake, their rate of weight gain and energy balance was comparable to that observed in the non-ETI group. This suggests that the higher prevalence of pancreatic insufficiency in the ETI group may have significantly contributed to this outcome. The results of our analysis do not provide compelling evidence that ETI therapy during a rehabilitation programme with regular exercise sessions leads to a disproportionate gain in body weight or fat mass.

## Figures and Tables

**Table 1:** Baseline characteristics of the study population.

Total Study Population	ETI (*n* = 38)	non-ETI (*n* = 16)	*p*-Value
Age, years	37 ± 1	42 ± 2	0.06
BMI, kg/m^2^	23.8 ± 0.3	24.7 ± 0.6	0.37
ppFEV1, %	68 ± 2	70 ± 3	0.75
Calculated REE ^a^, kcal/d	1511 ± 21	1588 ± 43	0.11
Exocrine Pancreatic Insufficiency	35 (92%)	8 (50%)	**<0.001**
Pseudomonas aeruginosa	24 (63%)	9 (56%)	0.65
ΔF508 homozygous	17 (45%)	3 (19%)	**0.002**
ΔF508 heterozygous	18 (47%)	7 (44%)	**0.002**
CFRD	15 (40%)	3 (19%)	0.06
**Women** (ETI: *n* = 22, non-ETI: *n* = 8)			
BMI, kg/m^2^	23.7 ± 0.5	24.6 ± 0.7	0.52
FMI, kg/m^2^	7.6 ± 0.4	8.4 ± 0.7	0.47
FFMI, kg/m^2^	16.1 ± 0.2	16.1 ± 0.2	0.92
**Men** (ETI: *n* = 16, non-ETI: *n* = 8)			
BMI, kg/m^2^	23.9 ± 0.4	24.8 ± 0.9	0.57
FMI, kg/m^2^	5.3 ± 0.3	5.1 ± 0.6	0.67
FFMI, kg/m^2^	18.6 ± 0.2	19.7 ± 0.5	0.10

Values are either mean ± SE or absolute number (%). *p*-values indicate differences between groups assessed by independent samples *t*-test (metric variables) or chi-squared test (categorical variables). Significant differences (*p* < 0.05) are indicated in bold. CFRD, cystic-fibrosis-related diabetes; ETI, Elexacaftor/Tezacaftor/Ivacaftor therapy; FMI, fat mass index; FFMI, fat-free mass index; non-ETI, no ETI or other CFTR modulator therapy; ppFEV1, percent predicted forced expiratory volume in one second; REE, resting energy expenditure ^a^ calculated according to Harris and Benedict (1918) [39].

**Table 2:** Comparison of T1–T0 changes in BMI, body composition, energy balance, and plasma leptin levels during the rehabilitation programme between ETI and non-ETI groups.

	ETI (*n* = 38)		non-ETI (*n* = 16)		*p*-Value
	mean ± SE	95% CI	mean ± SE	95% CI	ETI vs. non-ETI
Δ BMI, kg/m^2^	0.3 ± 0.1	0.1–0.5	0.3 ± 0.1	0.02–0.5	0.97
Energy Balance, kcal/ 3 weeks	4823 ± 2785	−814–10,460	3810 ± 3056	−2376–9996	0.82
**Women ** (ETI: *n* = 22, non-ETI: *n* = 8)					
Δ BMI, kg/m^2^	0.3 ± 0.1	0.1–0.6	0.3 ± 0.2	−0.1–0.6	0.86
Δ FMI, kg/m^2^	0.1 ± 0.1	−0.2–0.4	0.04 ± 0.2	−0.5–0.5	0.96
Δ FFMI, kg/m^2^	0.2 ± 0.1	−0.04–0.4	0.2 ± 0.2	−0.1–0.6	0.99
Energy Balance, kcal/ 3 weeks	9444 ± 3330	2702–16,186	4427 ± 3207	−2066–10,921	0.46
Δ Plasma Leptin, ng/mL ^a^	7.7 ± 2.7	2.1–13.3	17.1 ± 16.7	−17.7–52.0	0.82
**Men ** (ETI: *n* = 16, non-ETI: *n* = 8)					
Δ BMI, kg/m^2^	0.2 ± 0.1	−0.03–0.4	0.3 ± 0.2	−0.1–0.7	0.90
Δ FMI, kg/m^2^	−0.2 ± 0.1	−0.5–0.5	0.01 ± 0.2	−0.4–0.4	0.64
Δ FFMI, kg/m^2^	0.3 ± 0.1	0.1–0.6	0.3 ± 0.2	−0.2–0.8	0.99
Energy Balance, kcal/ 3 weeks	202 ± 3705	−7299–7703	3193 ± 5280	−7497–13,882	0.88
Δ Plasma Leptin, ng/mL ^a^	−0.02 ± 1.5	−3.1–3.1	3.1 ± 2.4	−2.0–8.2	0.53

Results are adjusted for training type and prevalence of exocrine pancreatic insufficiency. Δ indicates the change between T0 and T1. *p*-values refer to differences between ETI vs. non-ETI, assessed by contrast tests using a linear model. Energy balance was calculated from changes in body composition. ETI, Elexacaftor/Tezacaftor/Ivacaftor therapy; FMI, fat mass index; FFMI, fat-free mass index; non-ETI, no ETI or other CFTR modulator therapy; ^a^ n for plasma leptin, ETI/non-ETI: women: *n* = 18/*n* = 5; men: *n* = 11/*n* = 7.

## Data Availability

The data supporting this study’s findings are not publicly available due to the data privacy statement in the subject information form and are available from the corresponding author A.B.-W. upon reasonable request.

## Supplementary Materials

The following supporting information can be downloaded at: https://www.mdpi.com/article/10.3390/nu17152439/s1, Table S1: Missing data for outcome parameters due to organisational problems, unsuccessful blood draws, and incomplete questionnaires; Table S2: Comparison of T1-T0 changes in energy balance during the rehabilitation programme in subgroups of pwCF within ETI or non-ETI groups with or without exocrine pancreatic insufficiency.

## Abbreviations

The following abbreviations are used in this manuscript:

BMI	Body Mass Index
CF	Cystic Fibrosis
CFTR	Cystic Fibrosis Transmembrane Conductance Regulator
ETI	Elexacaftor, Tezacaftor, Ivacaftor
FFM	Fat-Free Mass
FFMI	Fat-Free Mass Index
FM	Fat Mass
FMI	Fat Mass Index
HIIT	High-Intensity Interval Training
MICT	Moderate-Intensity Continuous Training
PYY	Peptide YY
ppFEV1	Percent Predicted Forced Expiratory Volume in One Second
REE	Resting Energy Expenditure
SQ	Satiety Quotient
VAS	Visual Analogue Scale
VO2peak	Peak Oxygen Uptake
pwCF	People with Cystic Fibrosis

## References

[1] Elborn J.S. (2016). Cystic Fibrosis. Lancet.

[2] Naehrlich L., Burkhart M., Basler C., Dittrich A.-M., Ellemunter H., Hebestreit H., Nitsche O., Held I., Smaczny C., Sutharsan S. (2024). German Cystic Fibrosis Registry-Annual Report 2023.

[3] Bell S.C., Mall M.A., Gutierrez H., Macek M., Madge S., Davies J.C., Burgel P.-R., Tullis E., Castaños C., Castellani C. (2020). The Future of Cystic Fibrosis Care: A Global Perspective. Lancet Respir. Med..

[4] Sharma R., Florea V.G., Bolger A.P., Doehner W., Florea N.D., Coats A.J.S., Hodson M.E., Anker S.D., Henein M.Y. (2001). Wasting as an Independent Predictor of Mortality in Patients with Cystic Fibrosis. Thorax.

[5] European Medicines Agency Kalydeco. https://www.ema.europa.eu/en/medicines/human/EPAR/kalydeco#authorisation-details.

[6] Sutharsan S., Dillenhoefer S., Welsner M., Stehling F., Brinkmann F., Burkhart M., Ellemunter H., Dittrich A.-M., Smaczny C., Eickmeier O. (2023). Impact of Elexacaftor/Tezacaftor/Ivacaftor on Lung Function, Nutritional Status, Pulmonary Exacerbation Frequency and Sweat Chloride in People with Cystic Fibrosis: Real-World Evidence from the German CF Registry. Lancet Reg. Health Eur..

[7] Taylor-Cousar J.L., Robinson P.D., Shteinberg M. (2023). CFTR Modulator Therapy: Transforming the Landscape of Clinical Care in Cystic Fibrosis. Lancet.

[8] Bailey J., Rozga M., McDonald C.M., Bowser E.K., Farnham K., Mangus M., Padula L., Porco K., Alvarez J.A. (2021). Effect of CFTR Modulators on Anthropometric Parameters in Individuals with Cystic Fibrosis: An Evidence Analysis Center Systematic Review. J. Acad. Nutr. Diet..

[9] Heijerman H.G.M., McKone E.F., Downey D.G., Braeckel E.V., Rowe S.M., Tullis E., Mall M.A., Welter J.J., Ramsey B.W., McKee C.M. (2019). Efficacy and Safety of the Elexacaftor plus Tezacaftor plus Ivacaftor Combination Regimen in People with Cystic Fibrosis Homozygous for the F508del Mutation: A Double-Blind, Randomised, Phase 3 Trial. Lancet.

[10] European Medicines Agency Kaftrio. https://www.ema.europa.eu/en/medicines/human/EPAR/kaftrio#authorisation-details.

[11] Stallings V.A., Sainath N., Oberle M., Bertolaso C., Schall J.I. (2018). Energy Balance and Mechanisms of Weight Gain with Ivacaftor Treatment of Cystic Fibrosis Gating Mutations. J. Pediatr..

[12] Granados A., Chan C.L., Moheet A., Vigers T., Arbeláez A.M., Larson Ode K. (2023). The Impact of Elexacaftor/Tezacaftor/Ivacaftor on Body Composition in a Small Cohort of Youth with Cystic Fibrosis. Pediatr. Pulmonol..

[13] Grancini V., Gramegna A., Zazzeron L., Alicandro G., Porcaro L.L., Piedepalumbo F., Lanfranchi C., Daccò V., Orsi E., Blasi F. (2023). Effects of Elexacaftor / Tezacaftor / Ivacaftor Triple Combination Therapy on Glycaemic Control and Body Composition in Patients with Cystic Fibrosis-Related Diabetes. Diabetes Metab..

[14] Hevilla F., Porras N., Girón M.V., García-Olivares M., Padial M., Sánchez-Torralvo F.J., Olveira C., Olveira G. (2024). Impact of Elexacaftor-Tezacaftor-Ivacaftor Therapy on Body Composition, Dietary Intake, Biomarkers, and Quality of Life in People with Cystic Fibrosis: A Prospective Observational Study. Nutrients.

[15] Knott-Torcal C., Sebastián-Valles F., Girón Moreno R.M., Martín- Adán J.C., Jiménez-Díaz J., Marazuela M., Sánchez de la Blanca N., Fernández-Contreras R., Arranz-Martín A. (2023). A Prospective Study to Assess the Impact of a Novel CFTR Therapy Combination on Body Composition in Patients with Cystic Fibrosis with *F508del* Mutation. Clin. Nutr..

[16] Merino Sánchez-Cañete A., López Cárdenes C.M., Vicente Santamaría S., Gutiérrez Martínez J.R., Suárez González M., Álvarez Merino M., González Jiménez D. (2024). Increased Fat Mass and Obesity Risk after Elexacaftor-Tezacaftor-Ivacaftor Therapy in Young Adults with Cystic Fibrosis. Front. Nutr..

[17] Navas-Moreno V., Sebastian-Valles F., Rodríguez-Laval V., Knott-Torcal C., Marazuela M., de la Blanca N.S., Arranz Martín J.A., Girón R.M., Sampedro-Núñez M.A. (2024). Impact of CFTR Modulator Therapy on Body Composition as Assessed by Thoracic Computed Tomography: A Follow-up Study. Nutrition.

[18] Trost S.U., Harindhanavudhi T., Ankireddypalli A., Wang Q., Simrah A., Avula S., Moheet A. Elexacaftor/Tezacaftor/Ivacator Effect on Bone Density and Body Composition–A Retrospective Analysis. 2024, preprint. https://ssrn.com/abstract=4882738.

[19] Westhölter D., Haubold J., Welsner M., Salhöfer L., Wienker J., Sutharsan S., Straßburg S., Taube C., Umutlu L., Schaarschmidt B.M. (2024). Elexacaftor/Tezacaftor/Ivacaftor Influences Body Composition in Adults with Cystic Fibrosis: A Fully Automated CT-Based Analysis. Sci. Rep..

[20] Zamponi V., Cirilli N., Caporelli N., Fabrizzi B., Strappato M., Mignini E., Nicolai G., Nicolari A., Taus M. (2023). One-Year Assessment of Body Composition in Cystic Fibrosis Patients on Elexacaftor-Tezacaftor-Ivacaftor. Clin. Case Rep. Int..

[21] Gur M., Bar-Yoseph R., Hanna M., Abboud D., Keidar Z., Palchan T., Toukan Y., Masarweh K., Alisha I., Zuckerman-Levin N. (2023). Effect of Trikafta on Bone Density, Body Composition and Exercise Capacity in CF: A Pilot Study. Pediatr. Pulmonol..

[22] López Cárdenes C.M., Merino Sánchez-Cañete A., Vicente Santamaría S., Gascón Galindo C., Merino Sanz N., Tabares González A., Blitz Castro E., Morales Tirado A., Garriga García M., López Rozas M. (2024). Effects on Growth, Weight and Body Composition after CFTR Modulators in Children with Cystic Fibrosis. Pediatr. Pulmonol..

[23] Proud D., Duckers J. (2023). Weight a Minute: Exploring the Effect on Weight and Body Composition after the Initiation of Elexacaftor/Tezacaftor/Ivacaftor in Adults with CF. J. Cyst. Fibros..

[24] Sheikh S., Zemel B.S., Stallings V.A., Rubenstein R.C., Kelly A. (2014). Body Composition and Pulmonary Function in Cystic Fibrosis. Front. Pediatr..

[25] Wilschanski M., Munck A., Carrion E., Cipolli M., Collins S., Colombo C., Declercq D., Hatziagorou E., Hulst J., Kalnins D. (2024). ESPEN-ESPGHAN-ECFS Guideline on Nutrition Care for Cystic Fibrosis. Clin. Nutr..

[26] German Pension Insurance Concept for the Inpatient Rehabilitation of Children and Adolescents with Cystic Fibrosis. https://www.google.com/url?sa=t&source=web&rct=j&opi=89978449&url=https://www.deutsche-rentenversicherung.de/SharedDocs/Downloads/DE/Experten/infos_reha_einrichtungen/konzepte_systemfragen/konzepte/konzept_kinder_mukoviszidose.pdf%3F__blob%3DpublicationFile%26v%3D1&ved=2ahUKEwidqIrq1tuJAxU8BdsEHdqaALwQFnoECDMQAQ&usg=AOvVaw1cfIUZpydc_qHtvf26K3KL.

[27] Van Biervliet S., Declercq D., Dereeper S., Vermeulen D., Würth B., De Guschtenaere A. (2021). The Effect of an Intensive Residential Rehabilitation Program on Body Composition in Patients with Cystic Fibrosis. Eur. J. Pediatr..

[28] Swisher A.K., Hebestreit H., Mejia-Downs A., Lowman J.D., Gruber W., Nippins M., Alison J., Schneiderman J. (2015). Exercise and Habitual Physical Activity for People With Cystic Fibrosis: Expert Consensus, Evidence-Based Guide for Advising Patients. Cardiopulm. Phys. Ther. J..

[29] Blundell J.E., Gibbons C., Caudwell P., Finlayson G., Hopkins M. (2015). Appetite Control and Energy Balance: Impact of Exercise. Obes. Rev..

[30] Gruber W., Koop J., Haegele F.A., Falkenberg C., Dewey S., Weiser B., Bosy-Westphal A. (2025). Presentation WS03.02 High-Intensity Interval Training and Moderate-Intensity Continuous Training Are Equivalent in Improving Exercise Capacity at Submaximal Intensity in Adults with Cystic Fibrosis. J. Cyst. Fibros..

[31] Quanjer P.H., Stanojevic S., Cole T.J., Baur X., Hall G.L., Culver B.H., Enright P.L., Hankinson J.L., Ip M.S.M., Zheng J. (2012). Multi-Ethnic Reference Values for Spirometry for the 3-95-Yr Age Range: The Global Lung Function 2012 Equations. Eur. Respir. J..

[32] Godfrey S., Mearns M. (1971). Pulmonary Function and Response to Exercise in Cystic Fibrosis. Arch. Dis. Child..

[33] Hebestreit H., Arets H.G.M., Aurora P., Boas S., Cerny F., Hulzebos E.H.J., Karila C., Lands L.C., Lowman J.D., Swisher A. (2015). Statement on Exercise Testing in Cystic Fibrosis. Respiration.

[34] Flint A., Raben A., Blundell J.E., Astrup A. (2000). Reproducibility, Power and Validity of Visual Analogue Scales in Assessment of Appetite Sensations in Single Test Meal Studies. Int. J. Obes..

[35] Anderson G., Catherine N., Woodend D., Wolever T. (2002). Inverse Association between the Effect of Carbohydrates on Blood Glucose and Subsequent Short-Term Food Intake in Young Men. Am. J. Clin. Nutr..

[36] Beaulieu K., Oustric P., Alkahtani S., Alhussain M., Pedersen H., Quist J.S., Færch K., Finlayson G. (2020). Impact of Meal Timing and Chronotype on Food Reward and Appetite Control in Young Adults. Nutrients.

[37] Drapeau V., King N., Hetherington M., Doucet E., Blundell J., Tremblay A. (2007). Appetite Sensations and Satiety Quotient: Predictors of Energy Intake and Weight Loss. Appetite.

[38] Green S.M., Delargy H.J., Joanes D., Blundell J.E. (1997). A Satiety Quotient: A Formulation to Assess the Satiating Effect of Food. Appetite.

[39] Harris J.A., Benedict F.G. (1918). A Biometric Study of Human Basal Metabolism. Proc. Natl. Acad. Sci. USA.

[40] European Parliament and Council (2011). Regulation (EU) No 1169/2011 of the European Parliament and of the Council of 25 October 2011 on the Provision of Food Information to Consumers.

[41] Forbes G.B. (2000). Body Fat Content Influences the Body Composition Response to Nutrition and Exercise. Ann. N. Y Acad. Sci..

[42] Gilmore L.A., Ravussin E., Bray G.A., Han H., Redman L.M. (2014). An Objective Estimate of Energy Intake during Weight Gain Using the Intake-Balance Method. Am. J. Clin. Nutr..

[43] de Jonge L., DeLany J.P., Nguyen T., Howard J., Hadley E.C., Redman L.M., Ravussin E. (2007). Validation Study of Energy Expenditure and Intake during Calorie Restriction Using Doubly Labeled Water and Changes in Body Composition. Am. J. Clin. Nutr..

[44] Racette S.B., Das S.K., Bhapkar M., Hadley E.C., Roberts S.B., Ravussin E., Pieper C., DeLany J.P., Kraus W.E., Rochon J. (2012). Approaches for Quantifying Energy Intake and %calorie Restriction during Calorie Restriction Interventions in Humans: The Multicenter CALERIE Study. Am. J. Physiol. Endocrinol. Metab..

[45] Faul F., Erdfelder E., Lang A.-G., Buchner A. (2007). G*Power 3: A Flexible Statistical Power Analysis Program for the Social, Behavioral, and Biomedical Sciences. Behav. Res. Methods.

[46] Gruber W., Orenstein D.M., Braumann K.M., Beneke R. (2014). Interval Exercise Training in Cystic Fibrosis -- Effects on Exercise Capacity in Severely Affected Adults. J. Cyst. Fibros..

[47] R Core Team R: The R Project for Statistical Computing. https://www.r-project.org/.

[48] Carey V.J., Wang Y.-G. (2002). Mixed-Effects Models in S and S-Plus. J. Am. Stat. Assoc..

[49] Carroll R.J., Ruppert D. (1988). Transformation and Weighting in Regression.

[50] Ockenga J. (2009). Importance of Nutritional Management in Diseases with Exocrine Pancreatic Insufficiency. HPB.

[51] Bretz F., Hothorn T., Westfall P.H. (2011). Multiple Comparisons Using R.

[52] Hothorn T., Bretz F., Westfall P. (2008). Simultaneous Inference in General Parametric Models. Biom. J..

[53] WHO A Healthy Lifestyle-WHO Recommendations. https://www.who.int/europe/news-room/fact-sheets/item/a-healthy-lifestyle---who-recommendations.

[54] Kristidis P., Bozon D., Corey M., Markiewicz D., Rommens J., Tsui L.C., Durie P. (1992). Genetic Determination of Exocrine Pancreatic Function in Cystic Fibrosis. Am. J. Hum. Genet..

[55] Ramsey M.L., Li S.S., Lara L.F., Gokun Y., Akshintala V.S., Conwell D.L., Heintz J., Kirkby S.E., McCoy K.S., Papachristou G.I. (2023). Cystic Fibrosis Transmembrane Conductance Regulator Modulators and the Exocrine Pancreas: A Scoping Review. J. Cyst. Fibros..

[56] Stastna N., Kunovsky L., Svoboda M., Pokojova E., Homola L., Mala M., Gracova Z., Jerabkova B., Skrickova J., Trna J. (2024). Improved Nutritional Outcomes and Gastrointestinal Symptoms in Adult Cystic Fibrosis Patients Treated with Elexacaftor/Tezacaftor/Ivacaftor. Dig. Dis..

[57] Petersen M.C., Begnel L., Wallendorf M., Litvin M. (2022). Effect of Elexacaftor-Tezacaftor-Ivacaftor on Body Weight and Metabolic Parameters in Adults with Cystic Fibrosis. J. Cyst. Fibros..

[58] Martin C., Burnet E., Ronayette-Preira A., de Carli P., Martin J., Delmas L., Prieur B., Burgel P.-R. (2021). Patient Perspectives Following Initiation of Elexacaftor-Tezacaftor-Ivacaftor in People with Cystic Fibrosis and Advanced Lung Disease. Respir. Med. Res..

[59] Bailey J. (2021). Nutritional and Metabolic Effects of Elexacaftor/Tezacaftor/Ivacaftor in Adults and Adolescents with Cystic Fibrosis. Ph.D. Thesis.

[60] Caley L.R., Jarosz-Griffiths H.H., Smith L., Gale L., Barrett J., Kinsey L., Davey V., Nash M., Jones A.M., Whitehouse J.L. (2023). Body Mass Index and Nutritional Intake Following Elexacaftor/Tezacaftor/Ivacaftor Modulator Therapy in Adults with Cystic Fibrosis. J. Cyst. Fibros..

[61] Batterham R.L., Cowley M.A., Small C.J., Herzog H., Cohen M.A., Dakin C.L., Wren A.M., Brynes A.E., Low M.J., Ghatei M.A. (2002). Gut Hormone PYY(3-36) Physiologically Inhibits Food Intake. Nature.

[62] Litvin M., Yoon J.C., Leey Casella J., Blackman S.M., Brennan A.L. (2019). Energy Balance and Obesity in Individuals with Cystic Fibrosis. J. Cyst. Fibros..

[63] Minzoni A., Mazzetti L., Orlando P., Licci G., Taccetti G., Bresci S., Maggiore G. (2024). Cystic Fibrosis-Related Chronic Rhinosinusitis: The Key Role of a Comprehensive Evaluation in the Era of Highly Effective Modulator Therapy. Eur. Arch. Otorhinolaryngol..

[64] Tervo J.P., DiMango E., Gudis D.A., Keating C., Zhang Y., Leu C.-S., Altman K., Vilarello B., Jacobson P., Overdevest J.B. (2023). Olfaction, Body Mass Index, and Quality of Life with Cystic Fibrosis Combination Therapy. Int. Forum Allergy Rhinol..

[65] Bacon D.R., Stapleton A., Goralski J.L., Ebert C.S., Thorp B.D., Nouraie M., Shaffer A.D., Senior B.A., Lee S.E., Zemke A.C. (2022). Olfaction before and after Initiation of Elexacaftor-Tezacaftor-Ivacaftor in a Cystic Fibrosis Cohort. Int. Forum Allergy Rhinol..

[66] Beswick D.M., Humphries S.M., Balkissoon C.D., Strand M., Vladar E.K., Ramakrishnan V.R., Taylor-Cousar J.L. (2022). Olfactory Dysfunction in Cystic Fibrosis: Impact of CFTR Modulator Therapy. J. Cyst. Fibros..

[67] Halton T.L., Hu F.B. (2004). The Effects of High Protein Diets on Thermogenesis, Satiety and Weight Loss: A Critical Review. J. Am. Coll. Nutr..

[68] Barkeling B., Rössner S., Björvell H. (1990). Effects of a High-Protein Meal (Meat) and a High-Carbohydrate Meal (Vegetarian) on Satiety Measured by Automated Computerized Monitoring of Subsequent Food Intake, Motivation to Eat and Food Preferences. Int. J. Obes..

[69] Johnson J., Vickers Z. (1993). Effects of Flavor and Macronutrient Composition of Food Servings on Liking, Hunger and Subsequent Intake. Appetite.

[70] Weigle D.S., Breen P.A., Matthys C.C., Callahan H.S., Meeuws K.E., Burden V.R., Purnell J.Q. (2005). A High-Protein Diet Induces Sustained Reductions in Appetite, Ad Libitum Caloric Intake, and Body Weight despite Compensatory Changes in Diurnal Plasma Leptin and Ghrelin Concentrations. Am. J. Clin. Nutr..

[71] Fazzino T.L., Courville A.B., Guo J., Hall K.D. (2023). Ad Libitum Meal Energy Intake Is Positively Influenced by Energy Density, Eating Rate and Hyper-Palatable Food across Four Dietary Patterns. Nat. Food.

[72] Bosy-Westphal A., Hägele F.A., Müller M.J. (2021). Impact of Energy Turnover on the Regulation of Energy and Macronutrient Balance. Obesity.

[73] Prévotat A., Godin J., Bernard H., Perez T., Le Rouzic O., Wallaert B. (2019). Improvement in Body Composition Following a Supervised Exercise-Training Program of Adult Patients with Cystic Fibrosis. Respir. Med. Res..

[74] Gruet M., Saynor Z.L., Urquhart D.S., Radtke T. (2022). Rethinking Physical Exercise Training in the Modern Era of Cystic Fibrosis: A Step towards Optimising Short-Term Efficacy and Long-Term Engagement. J. Cyst. Fibros..

[75] Radtke T., Smith S., Nevitt S.J., Hebestreit H., Kriemler S. (2022). Physical Activity and Exercise Training in Cystic Fibrosis. Cochrane Database Syst. Rev..

[76] Taelman V., Declercq D., Van Biervliet S., Weygaerde Y.V., Lapauw B., Van Braeckel E. (2023). Effect of 18 months elexacaftor-tezacaftor-ivacaftor on body mass index and glycemic control in adults with cystic fibrosis. Clin. Nutr. ESPEN.

