# Supplementary material for: A 3-Week Inpatient Rehabilitation Programme Improves Body Composition in People with Cystic Fibrosis with and Without Elexacaftor/Tezacaftor/Ivacaftor Therapy
Source: Nutrients. 2025 Jul 25;17(15):2439. doi: 10.3390/nu17152439 (PMC12348923; doi:10.3390/nu17152439)
Supplement: Supplementary file 1 [file nutrients-17-02439-s001.zip › nutrients-3728331-supplementary.pdf]

Supplementary Material

Table S1. Missing data for outcome parameters due to organisational problems, unsuccessful blood draws and incomplete questionnaires.

	Total	ETI	non-ETI
Body Composition	4	3	1
Meal Energy Intake	21	16	5
Appetite-regulating Hormones	22	17	5
Subjective Appetite	25	19	6
24-h Energy Intake	27	20	7

Table S2. Comparison of T1-T0 changes in energy balance during the rehabilitation programme in subgroups of pwCF within ETI or non-ETI groups with or without exocrine pancreatic insufficiency.

	**Pancreatic sufficient**		**Exocrine pancreatic insufficency**		**p-value**
	mean ±SE	95% CI	mean ±SE	95% CI	
**ETI** (n=3, n=35)					
Energy balance, kcal/3 weeks	9,768 ±4,399	862-18,674	-122 ±2,178	-4,532-4,288	**0.01**
**non-ETI** (n=8, n=8)					
Energy balance, kcal/3 weeks	8,755 ±3,449	1,774-15,737	-1,135 ±3,920	-9,072-6,801	**0.01**

Results are adjusted for training type and sex. p-values refer to differences between pwCF with and without exocrine pancreatic insufficiency within ETI and non-ETI groups, assessed by contrast tests using a linear model. Energy balance was calculated from changes in body composition. ETI, Elexacaftor/Tezacaftor/Ivacaftor therapy; non-ETI, no ETI or other CFTR modulator therapy; pwCF, people with CF.
